# Estimated rate of agricultural injury: the Korean Farmers’ Occupational Disease and Injury Survey

**DOI:** 10.1186/2052-4374-26-8

**Published:** 2014-04-25

**Authors:** Hyeseon Chae, Kyungdoo Min, kanwoo Youn, Jinwoo Park, Kyungran Kim, Hyocher Kim, Kyungsuk Lee

**Affiliations:** 1 National Academy of Agricultural Science, Rural Development Administration, Suwon, Republic of Korea; 2 Wonjin Institute for Occupational and Environmental Health, Seoul, Republic of Korea; 3 Department of Applied Statistics, University of Suwon, Suwon, Republic of Korea

**Keywords:** Agriculture, Farmer, Occupational injury, Disease, Accident, Statistics, Injury rate

## Abstract

**Objectives:**

This study estimated the rate of agricultural injury using a nationwide survey and identified factors associated with these injuries.

**Methods:**

The first Korean Farmers’ Occupational Disease and Injury Survey (KFODIS) was conducted by the Rural Development Administration in 2009. Data from 9,630 adults were collected through a household survey about agricultural injuries suffered in 2008. We estimated the injury rates among those whose injury required an absence of more than 4 days. Logistic regression was performed to identify the relationship between the prevalence of agricultural injuries and the general characteristics of the study population.

**Results:**

We estimated that 3.2% (±0.00) of Korean farmers suffered agricultural injuries that required an absence of more than 4 days. The injury rates among orchard farmers (5.4 ± 0.00) were higher those of all non-orchard farmers. The odds ratio (OR) for agricultural injuries was significantly lower in females (OR: 0.45, 95% CI = 0.45–0.45) compared to males. However, the odds of injury among farmers aged 50–59 (OR: 1.53, 95% CI = 1.46–1.60), 60–69 (OR: 1.45, 95% CI = 1.39–1.51), and ≥70 (OR: 1.94, 95% CI = 1.86–2.02) were significantly higher compared to those younger than 50. In addition, the total number of years farmed, average number of months per year of farming, and average hours per day of farming were significantly associated with agricultural injuries.

**Conclusions:**

Agricultural injury rates in this study were higher than rates reported by the existing compensation insurance data. Males and older farmers were at a greater risk of agriculture injuries; therefore, the prevention and management of agricultural injuries in this population is required.

## Introduction

Occupational injuries in the agricultural sector have been reported to be higher than the average accident rate in all other industries taken together [1,2]. Most farmers who become physically impaired due to agricultural work are then temporarily or permanently limited in their ability to perform further agricultural activities [3] and loss to productivity and income is estimated to reach about 5.2 trillion Korean won [4]. The rate of occupational accidents among farmers has been rising in the Republic of Korea (hereafter, “Korea”); therefore, an accident compensation system that protects workers from accidents and disease related to agricultural work is being developed. With the goal of creating evidence-based policies, accurate data are needed to understand the scale and etiology behind occupational injuries in agricultural farm work.

Until now, the rate of agricultural injuries in Korea has been identified using mostly insurance claims data. However, these data do not accurately represent all of the agricultural injuries in Korean farmers because only approved data on convalescence are used to calculate the rate of agricultural injuries. In addition, the injuries of self-employed farmers are not submitted to this type of insurance claims data, and thus are fundamentally excluded from the calculation process. Moreover, other injuries reported in the safety aid system are typically excluded from the insurance claims data, and thus are also not included in the agricultural injury calculation. Therefore, the scale of agricultural injuries may be underestimated.

In Korea, statistical research into agricultural injuries is necessary to accurately identify the scope and prevalence of these injuries. This research can then be used as an educational resource or to develop policy interventions and programs. In the Labour Force Survey from the UK [5] and the Occupational Injury Surveillance of Production Agriculture Survey from the US [3], data were collected from a nationally representative sample of farmers. However, researchers in the US also conducted a regional survey called the Farm Health Interview Survey [3,6]. In Korea, the Korean Farmers’ Occupational Disease and Injury Survey (KFODIS) was promoted by the Rural Development Administration from 2009. Previous agriculture injury studies conducted in Korea only investigated injuries associated with farm equipment/machinery [7-10], and most of these studies were only performed in rural areas [7-9,11]. To collect representative data on the agricultural injury status among Korean farmers, one previous study traced the development of monitoring systems and the monitoring of the incidence, cause, treatment, and outcomes of agricultural injuries [12]. In addition, efforts have been made to prepare a statistical plan to monitor agricultural injuries [13]. However, there are some limitations in using these data to predict and understand agricultural injuries among Korean farmers because the populations used were not representative and limited resources were available for the statistical analyses.

Therefore, we aimed to estimate the agricultural injury rate among Korean farmers using data from the first KFODIS to identify the relationship between agricultural injury and general characteristics such as gender, age, total experience farming, average number of months per year of farming, and average number of hours per day of farming.

## Materials and methods

### Study subjects

Beginning in 2009, the Rural Development Administration performed the KFODIS to understand the scope of agricultural injuries and develop evidence-based management policies.

All farms within *dongs* (the smallest administrative district in urban areas), *eups*, and *myeons* (the smallest districts in rural areas) were included in the survey population; however, islands with <100 farms or <20% of farms per area were excluded from the survey population.

Multi-stage stratified sampling was conducted using three sampling units from 2005 agriculture census data. The first sampling unit included *dongs*, *eups*, and *myeons*, and the second sampling unit included smaller villages within *dongs*, *eups*, *myeons*, or towns. The third sampling unit included households in the villages. The number of farms, agricultural population by age, and percentage of apartments were used as stratification variables.

The study sample was determined from 6,000 households corresponding to a coefficient of variation of 5–6% according to the agricultural machinery accident rates from the Rural Life Indicators Survey in 2008. In total, 400 villages were determined as survey areas, and 15 households per survey area were selected to participate in the KFODIS. All members of these households over the age of 19 were eligible for the KFODIS.

### Methods

In previous studies, agricultural injury was defined as physical injuries due to falling, traffic accidents during farm work, careless farm machine operation, collisions [11], or any injury/disease caused by agricultural work [12,14]. In the KFODIS, agricultural injury was defined as accidents resulting during preparation, performance, cleanup, or movements required by farm work. Data were collected from January 1 to December 31 of the year (2008) before the survey.

The 2009 questionnaire was the first KFODIS and consisted of a main (20 items) and supplemental questionnaire (9 items). The main questionnaire collected data on the general characteristics of famers and any experiences related to agricultural injury. The supplemental questionnaire collected detailed information on any of their reported agricultural injuries.

The variables used in our analysis from the main questionnaire were collected about each household and included the participant’s age, gender, types of farming, total years farming, average number of months per year of farming, average hours per day of farming, and any incidents that caused an agricultural injury. In the supplemental questionnaire, data on the number of absences due to injury were also collected. Answering “yes” to whether an agriculture-related accidents or poisoning had occurred in the main questionnaire was used to define any farmers who experienced agricultural injuries. Among those who had experienced an agricultural injury, the number of days not spent working due to their injury was defined as ≥4 days.

### Data collection

To increase the outreach of our data collection, 322 people residing in the 400 sampled villages were recruited as interviewers. Interviewers were trained on the purpose of survey, definition of injury, relevant farm work, investigation guidelines, and other important areas. We expected that most survey participants would tend to be older and less educated than the general population of Korea; therefore, the skills and techniques of the interviewers were deemed important. The managers of the KFODIS visited the main cities and counties to train all of the interviewers of the surrounding 2–6 villages. If the surveyor selected did not participate in the survey, the interviewer in the neighborhood was responsible for the survey of the area. Survey errors were minimized by providing specific details and instructions to all interviewers. In addition, before collecting data, the purpose of the survey was fully explained to all participants and informed consent was obtained. Surveyors visited sampled households from November 1 to 30, 2009, and 4,809 households of 6,000 sampled households were surveyed (80.2% response rate). In total, data on 9,630 farmers were collected.

### Data analysis

After excluding those with missing data, 8,064 people were eligible for our analysis. Of these, people who reported experiencing agricultural injuries with more than 4 days of absence were finally selected and made up our study population.

Sampling weights were applied by calculating a sample extraction probability, non-response adjustment, post-stratification process, as well as individual sample weights. SPSS PC + (ver. 19.0) was used for all data analyses. In addition, the frequency and percentage of the study population’s general characteristics were compared to the rates of injury according to the type of agriculture using the Rao-Scott chi-squared test. Logistic regression analyses were also performed to determine factors related to agricultural injury.

## Results

### General characteristics of the study population

Of the 8,064 total participants, 4,324 (53.6%) were males and 3,740 (46.4%) were females. Those aged 60–69 years old (30.5%) and ≥70 years (30.4%) accounted for 60.9% of the all those surveyed followed by those aged 50–59 years (25.2%) and <50 years (13.9%). For the total years each participant had farmed, the majority had farmed for ≥20 years (84.1%), and the minority had farmed for <10 years (6.7%). Throughout the previous year, most participants had farmed ≥10 months (47.9%) followed by 7–9 months (31.4%) and <7 months (20.7%). In addition, most participants farmed 5–9 hours (59.8%) each day followed by <5 hours (25.5%). Those farming ≥10 hours were the minority (14.7%) (Table 1).

**Table 1:** General characteristics of the study population

**Variables**	**Number (%)**
	8,064 (100.0)
Gender	
Male	4,324 (53.6)
Female	3,740 (46.4)
Age (years)	
<50	1,124 (13.9)
50-59	2,030 (25.2)
60-69	2,460 (30.5)
≥70	2,450 (30.4)
Total years farming (years)	
<10	542 (6.7)
10-19	742 (9.2)
≥20	6,780 (84.1)
Average months per year of farming (months)	
<7	1,667 (20.7)
7-9	2,533 (31.4)
≥10	3,864 (47.9)
Average hours per day of farming (hours)	
<5	2,055 (25.5)
5-9	4,825 (59.8)
≥10	1,184 (14.7)

### Agricultural injury rate

Of 8,064 responses from the first KFODIS in 2009, 269 farmers suffered an injury that required more than 4 days of absence during the previous year (2008), and the estimated agricultural injury rate was 3.2% (±0.00, 95% confidence interval).

Statistically significant differences were found when estimating the agricultural injury rate according to each general characteristic of the study population. The injury rate among males (4.3% ± 0.00) was higher than that of females (1.9% ± 0.00). When we analyzed the data by age group, we found the injury rate of those ≥70 years to be the highest at 4.0% (±0.00) followed by those 50–59 years old (3.2% ± 0.00), 60–69 years old (3.1% ± 0.00), and <50 years old (1.9% ± 0.01). In addition, the injury rate among those with ≥20 years of experience was the highest at 3.5% (±0.00). Those with <10 years and 10–19 years of experience had an injury rate of 2.0% (±0.00) and 1.8% (±0.00), respectively. The injury rate according to the average number of months worked per year was highest for those working ≥10 months (4.1% ± 0.01), but was lower for those working 7–9 months (3.0% ± 0.01) and <7 months (1.5% ± 0.00). Similarly, the injury rate was the highest among those who worked ≥10 hours per day (4.7% ± 0.00) followed by those working 5–9 hours (3.5% ± 0.01) and <5 hours (1.7% ± 0.00) (Table 2).

**Table 2:** Estimated rate of agricultural injury by farmers’ characteristics

**Variables**	**Unweighted**		**Weighted**			
	N*	**n**^**†**^	**N**^**‡**^	**n**^**§**^	**%±SE**^**∥**^	**p-value**^**⁋**^
	8064	269	1,661,064	53,301	3.2±0.00	
Gender						
Male	4,324	195	916,683	39,300	4.3±0.00	0.001
Female	3,740	74	744,382	14,001	1.9±0.00	
Age (years)						
<50	1,124	21	217,876	4,043	1.9±0.01	0.003
50-59	2,030	66	410,701	13,046	3.2±0.00	
60-69	2,460	80	517,046	15,840	3.1±0.00	
≥70	2,450	102	515,441	20,372	4.0±0.00	
Total years farming (years)						
<10	542	10	106,369	2,171	2.0±0.00	0.001
10-19	742	14	152,987	2,678	1.8±0.00	
≥20	6,780	245	1,401,709	48,452	3.5±0.00	
Average months per year of farming (months)						
<7	1,667	28	350,121	5,103	1.5±0.00	0.002
7-9	2,533	82	503,324	15,027	3.0±0.01	
≥10	3,864	159	807,619	33,170	4.1±0.01	
Average hours per day of farming (hours)						
<5	2,055	38	411,985	7,084	1.7±0.00	0.001
5-9	4,825	175	1,017,031	35,409	3.5±0.01	
≥10	1,184	56	232,048	10,807	4.7±0.00	

*Number of farmers who participated in the survey per category.

^†^Unweighted number of farmers who reported an agricultural injury that required an absence of more than 4 days.

^‡^Estimated number of farmers in each category.

^§^Estimated number of farmers who reported an agricultural injury that required an absence of more than 4 days for each category.

^∥^Standard error.

^⁋^p-value calculated by Rao-Scott’s chi-squared test.

### Agricultural injury rates according to the type of farming

The type of farming was categorized as rice, dry fields, orchards, greenhouses, and livestock and the rate of agricultural injury was estimated for each type of farming. When compared to all those not farming in each category, the injury rates of orchard farmers were the highest at 5.4% (±0.00) followed by greenhouse at 3.6% (±0.02), dry fields at 3.3% (±0.00), and livestock farmers at 3.4% (±0.00%). However, no differences were found for rice farmers (Table 3).

**Table 3:** Estimated rate of agricultural injury by farm type

**Variables**	**Unweighted**		**Weighted**			
	N*	**n**^**†**^	**N**^**‡**^	**n**^**§**^	**%±SE**^**∥**^	**p-value**^**⁋**^
	8,064	269	1,661,064	53,301	3.2±0.00	
Rice farming						
Cultivation	6,950	232	1,431,449	45,770	3.2±0.00	0.060
No cultivation	1,114	37	229,615	7,530	3.3±0.01	
Dry fields farming						
Cultivation	6,524	224	1,331,556	44,533	3.3±0.00	0.004
No cultivation	1,540	45	329,508	8,768	2.7±0.00	
Orchard farming						
Cultivation	1,359	70	309,554	16,794	5.4±0.00	0.001
No cultivation	6,705	199	1,351,510	36,506	2.7±0.00	
Greenhouse farming						
Cultivation	1,736	66	341,656	12,466	3.6±0.02	0.021
No cultivation	6,328	203	1,319,408	40,834	3.1±0.00	
Livestock farming						
Cultivation	2,916	99	596,416	20,370	3.4±0.00	0.001
No cultivation	5,148	170	1,064,648	32,931	3.1±0.00	

^*^Number of farmers who participated in the survey per category.

^†^Unweighted number of farmers who reported an agricultural injury that required an absence of more than 4 days.

^‡^Estimated number of farmers in each category.

^§^Estimated number of farmers who reported an agricultural injury that required an absence of more than 4 days for each category.

^∥^Standard error.

^⁋^p-value calculated by Rao-Scott’s chi-squared test.

### Relationship between farmers’ general characteristics and occupational injury

The results of the Rao-Scott’s chi-squared test are shown in Table 2. The results of the logistic regression analyses revealed that the odds ratio (OR) and 95% confidence interval (CI) for agricultural injury was significantly reduced in females (OR: 0.45) (95% CI 0.45–0.45) when compared to males. Compared to those <50 years of age, those ≥70 years (OR: 1.94) (95% CI 1.86–2.02), 50–59 years (OR: 1.53) (95% CI 1.46–1.60), and 60–69 years (OR: 1.45) (95% CI 1.39–1.51) had a significantly increased risk of agricultural injury. In addition, those farming for 10–19 years (OR: 0.76) (95% CI 0.76–0.76) had a significantly lower odds of injury than that of those with <10 years of experience. However, those with ≥20 years of experience had an OR of 1.16 (95% CI 1.14–1.18). Moreover, the OR of injury among those with ≥10 months of work per year was 2.24 (95% CI 2.20–2.28) when compared to those working <7 months. Those working ≥10 hours per day were also at an increased odds of injury (OR: 1.79 (95% CI 1.77–1.82) compared to those working <5 hours per day (Table 4).

**Table 4:** Relationship between the general characteristics of farmers and agricultural injury

**Variables**	**OR**^**†**^	**95% CI**^**‡**^
Gender		
Male	1.00	
Female	0.45	0.45–0.45
Age (years)		
<50	1.00	
50-59	1.53	1.46–1.60
60-69	1.45	1.39–1.51
≥70	1.94	1.86–2.02
Total years farming (years)		
<10	1.00	
10-19	0.76	0.76–0.76
≥20	1.16	1.14–1.18
Average number of months per year of farming (months)		
<7	1.00	
7-9	1.70	1.67–1.74
≥10	2.24	2.20–2.28
Average hours per day of farming (hours)		
<5	1.00	
5-9	1.41	1.41–1.42
≥10	1.79	1.77–1.82

Adjusted for sex in the model.

^†^odds ratio, ^‡^95% confidence interval.

## Discussion

In this study, data related to agricultural injuries from the 2009 KFODIS were used. Through analysis of this data, which required an agricultural injury-related absence of more than 4 days, we estimated the rate of agricultural injury and revealed agricultural injury-related factors.

However, careful consideration should be made when attempting to compare our study to the existing injury rates published by the Industrial Accident Compensation Insurance and Safety Aid System of Farm Workers. For the data from the Industrial Accidents Compensation Insurance, only industrial accidents that occurred under the guidelines of the Industrial Accident Compensation Insurance Act were considered. In addition, these data only include industrial accidents related to disasters that require more than 4 days of absence, which had to have been documented as occupational accidents. These occupational accidents must also have been reported to the local employment labor offices. The Safety Aid Systems of Farm Workers collects data on accidents occurring during farm work that require more than 4 days of hospitalization. There is a difference between these two organizations in that certain infectious diseases or outpatient treatments are not included in the Industrial Accident Compensation Insurance. Therefore, the definitions of agricultural injury and the characteristics of the survey groups in this study and the previous ones should be considered different.

In this study, an estimated injury rate of 3.2% was calculated for injuries requiring more than 4 days of absence. However, the Industrial Accidents Status, which uses data from the Industrial Accidents Compensation Insurance, includes data on both injury and disease [1]. They calculated the total industrial injury rates and the agriculture injury rates in 2008 to be 0.68% and 1.35%, respectively. In addition, the agricultural injury rate according to the insurance claims data of the 2005 Safety Aid System of Farm Workers was 1.67% [14], which is similar to the injury rate calculated based on compensation insurance data, but was less than the injury rates in this study.

Another limitation of the Industrial Accident Compensation Insurance data is that most self-employed farmers were not included because they are not eligible for the accident insurance. However, the Safety Aid System of Farm Workers included data on self-employed farmers, but their data only contain information on compensation provided for accidental injuries. This may be the reason that the rate of agricultural injury calculated by the compensation insurance data was lower than that of this study.

According to the Labour Force Survey from the UK, the rate of agricultural injury was 3-6% for injuries requiring an absence of more than 3 days, even across different years [5]. Although it is difficult to compare their results with the results of our study, the distributions of the rate of agricultural injury show a similar pattern.

According to findings from the Korea National Health and Nutrition Survey, the rate of all occupational accidental injuries that required an absence of more than 4 days was 1.7% [2]. When these data were categorized according to each occupational cluster, the injury rates among skilled agricultural workers, including those with injuries requiring an absence of less than 4 days, was 4.1%. However, the injury rates for workers in the field of agriculture, forestry, and fishery were not present. Therefore, it is difficult to compare these findings with those of our study. In addition, it should be noted that the definition of injuries in the Korea National Health and Nutrition Survey was limited to those who had undergone medical treatment in hospitals or emergency rooms. Therefore, the scale of injuries may be smaller than that in our study.

A lack of sufficient data in our study may have limited our ability to measure injury rates among Korean farmers. Therefore, the injury rates estimated by compensation insurance data may have greater value than those estimated by a survey such as the one used in our study. In addition, the data used in our study may be subject to recall bias; thus, minor injuries may have been underreported.

We found the agricultural injury rates among orchard farmers to be the highest when we divided the types of farming into rice, dry fields, orchards, greenhouses, and livestock. One explanation for this finding may be that accidents such as falling are very likely to occur because crops (fruit trees) are tall; therefore, the position and environmental characteristics may affect the rate of injury. Since most Korean farmers engage in various types of agriculture, it is difficult to distinguish the influence of the type of farming on agricultural injury. However, future studies should consider how major crops or types of agricultural work might affect injuries by surveying participants about the proportion of operations of each agriculture type.

When we analyzed the relationship between gender and agricultural injury, males had a significantly higher OR of occupational injury than females, and the survey results of both Korea [14,7-11] and other countries [3,15-18] confirm these findings. The risk of injury among males may be higher than that among females because males may tend to use farm equipment more often or conduct more high-risk operations than females tend to do.

Looking at the relationship between age and injury, those 60–69 years old and those ≥70 years old had a 1.45 and 1.94 times higher odds of injury than did those <50 years old, respectively. In data from the Safety Aid System of Farm Workers [14] and those from other countries [3,15,19], the injury rates among those older than 60 years tended to be higher than that of those younger than 60. Moreover, in a study of emergency room patients suffering from agricultural injuries, those ≥60 years account for 40.4% of all patients who visited the emergency room for agricultural injuries [20]. These results suggest that issues related to work safety among older farmers may be a serious problem in many countries.

Having more experience (10–19 years) working on a farm revealed a decreased odds of 0.76 when compared with having <10 years of experience; however, having ≥20 years of experience was associated with a 1.16 times higher risk of injury than those with less experienced. This finding may be associated with increased age among the most experienced farmers.

The number of people in Korea who were engaged in the agriculture sector decreased sharply from 14,421,730 people in 1970 to 3,062,956 in 2010. As of 2009, 48.8% of the agricultural population was ≥60 years. In addition, aging among the farming population is an increasing trend [21]. With this aging, the risks of accidental injury increase as working conditions change and farmers are exposed to the various, uncontrollable factors of the natural environment. As a result, the increased age of farmers may be a stronger risk factor for agricultural injury than experience and skill level are. Therefore, the work environment for older farmers should be improved through work safety guidelines.

We found that working ≥10 months per year had a 2.24 times higher odds of injury than did working <7 months per year. In addition, working ≥10 hours per day had a 1.79 times higher odds of injury than did working <5 hours per day. The Korea National Health and Nutrition Survey also reported that there is an increased risk of injury as farmer worked more hours [2].

The limitations of this study are as follows. First, factors related to agricultural injury were identified through only basic characteristics of the population. Second, data collection may have been affected by recall bias since injuries were collected based on respondents’ memories.

Despite these limitations, this study is the first attempt at collecting data on agricultural injury in Korea. In addition, we identified the rate of agricultural injury among Korean farmers and found gender, age, the years of experience in farming, the average number of months farmed per year, and the average hours farmed per day to be associated factors.

Future studies are needed to develop a variety of resources and survey methods that can further identify the scale and characteristics of agricultural injury and its related factors. These findings are preliminary data on agricultural injury in Korea.

## Competing interests

The authors declare that they have no competing interests.

## Authors’ contributions

HSC, KSL, KWY, and JWP designed the study. KWY, JWP, and HSC collected the data. HSC and KDM performed the statistical analyses. All authors interpreted the data, wrote the manuscript, and read and approved the final manuscript.
